# Targeting hypoxic tumour cells to overcome metastasis

**DOI:** 10.1186/1471-2407-11-504

**Published:** 2011-11-30

**Authors:** Kevin L Bennewith, Shoukat Dedhar

**Affiliations:** 1 Integrative Oncology Department, British Columbia Cancer Agency, Vancouver, British Columbia, Canada

## Abstract

The microenvironment within solid tumours can influence the metastatic dissemination of tumour cells, and recent evidence suggests that poorly oxygenated (hypoxic) cells in primary tumours can also affect the survival and proliferation of metastatic tumour cells in distant organs. Hypoxic tumour cells have been historically targeted during radiation therapy in attempts to improve loco-regional control rates of primary tumours since hypoxic cells are known to be resistant to ionizing radiation-induced DNA damage. There are, therefore, a number of therapeutic strategies to directly target hypoxic cells in primary (and metastatic) tumours, and several compounds are becoming available to functionally inhibit hypoxia-induced proteins that are known to promote metastasis. This mini-review summarizes several established and emerging experimental strategies to target hypoxic cells in primary tumours with potential clinical application to the treatment of patients with tumour metastases or patients at high risk of developing metastatic disease. Targeting hypoxic tumour cells to reduce metastatic disease represents an important advance in the way scientists and clinicians view the influence of tumour hypoxia on therapeutic outcome.

## Review

The cellular environment within solid tumours is increasingly being appreciated as an important limitation to current cancer therapy. The vasculature within most solid tumours consists of abnormally formed, poorly functional blood vessels that are incapable of delivering sufficient oxygen and nutrients to properly support the growing tumour mass [1]. Available oxygen is consumed by rapidly proliferating tumour cells located within 70 to 150 μm of tumour vasculature, thereby limiting the amount of oxygen that is available to diffuse further into the tumour tissue. Thus, a proportion of cells in most tumours (ranging from < 1% to > 50%) are exposed to relatively low oxygen tensions (pO_2 _< 10 mmHg, equivalent to < 1.3% O_2 _*in vitro*). While reduced oxygen tensions can be lethal for some cells, many tumour cells are able to survive under poorly oxygenated (hypoxic) conditions. It is well-established that hypoxic tumour cells are resistant to radiation therapy, but the clinical impact of hypoxic tumour cells extends beyond the treatment of localized primary tumours with ionizing radiation. Hypoxic tumour cells promote tumour progression and metastasis through a variety of direct and indirect mechanisms, and hypoxic tumour cells, therefore, represent a significant impediment to successful cancer therapy.

**Figure 1:**
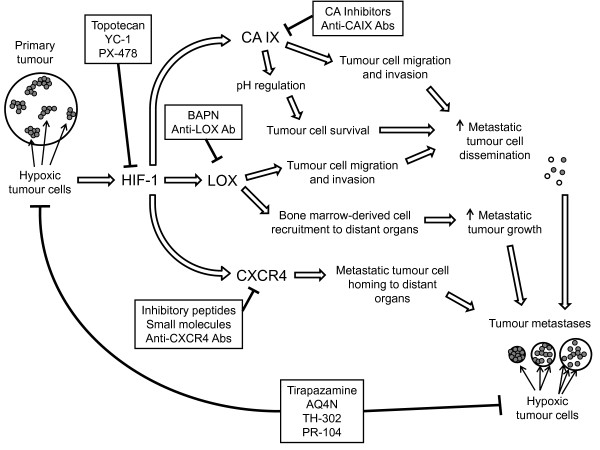
**Targeting tumour hypoxia to overcome metastasis**. Functional inhibition of the HIF-1 transcription factor or hypoxia-inducible proteins, such as CAIX, LOX, or CXCR4, can affect multiple steps in the metastatic process. A range of hypoxia-activated cytotoxins are also available to directly target hypoxic cells in primary tumours and in distant tumour metastases.

Patient survival rates are closely associated with the development of distant metastatic disease [2-4], with an estimated 90% of cancer-related deaths being attributed to the metastatic spread of cancer [5,6]. Patients with primary tumours that contain high proportions of hypoxic cells have decreased disease-free and overall survival rates after surgical resection of the primary tumour [7,8]. The decreased survival is due to the development of metastatic disease, suggesting that (undetected) disseminated tumour cells were present in the patient at the time of surgery. The strong association between the development of metastatic disease and the proportion of hypoxic cells in primary tumours suggests that hypoxic tumour cells promote a more aggressive, metastatic tumour phenotype. Indeed, hypoxia up-regulates over 80 genes associated with tumour progression, glycolysis, angiogenesis, and metastasis [9-12] through the transcriptional activity of the heterodimeric transcription factors hypoxia-inducible factor-1 (HIF-1) and HIF-2. The importance of hypoxia-induced genes in promoting metastatic tumour cell invasion and migration is well-established [12-17], and emerging evidence indicates that secreted hypoxia-induced proteins such as lysyl oxidase (LOX) [18] can modulate the microenvironment within distant metastatic target organs to promote metastatic tumour growth [19]. The relationship between tumour hypoxia and metastasis suggests that hypoxic tumour cells are key drivers of the metastatic process. A wide variety of systemic therapeutic strategies to directly target hypoxic tumour cells have been clinically tested in combination with radiation therapy in order to improve loco-regional control of primary tumours, and there is a clear need for new and efficacious strategies to treat (or manage) metastatic disease. We postulate that targeting hypoxic cells in primary tumours and metastatic tumour foci, as well as therapeutically inhibiting metastasis-associated proteins expressed by hypoxic tumour cells, are plausible therapeutic strategies to overcome tumour metastases that warrant further clinical investigation (Figure 1).

### Inhibition of hypoxia-induced metastasis-associated proteins

Hypoxic tumour cells are known to up-regulate a number of genes that promote metastasis. Consequently, the therapeutic inhibition or functional targeting of hypoxia-induced proteins holds promise as a potential strategy to decrease metastases in patients with hypoxic tumours. There are a number of small molecule inhibitors of HIF-1α (the hypoxia-responsive α-subunit of HIF-1) that have been identified [20,21] including topotecan [22], YC-1 [23], and PX-478 [24]. While therapeutic inhibition of HIF-1α has the potential to reduce the expression of a range of HIF-1 target genes, small molecule inhibition of transcription factors *in vivo *is inherently difficult and the tumour-specificity of HIF-1α inhibition is not clear. Thus, the inhibition of metastasis-associated proteins induced by hypoxia may provide more specific effects on metastatic tumour cell dissemination, metastatic tumour cell homing to distant organs, and metastatic tumour growth compared to HIF-1α inhibition, and several intriguing targets have been identified that hold promise for treating metastatic disease.

Carbonic anhydrase-9 (CAIX) is a hypoxia-induced cell surface protein involved in the regulation of intracellular pH. Therapeutic inhibition of CAIX has recently been shown to decrease primary tumour growth and metastasis in pre-clinical breast tumour models [25], partly by decreasing the ability of hypoxic tumour cells to adapt to the low extracellular pH found in hypoxic regions of primary tumours. A number of small molecule inhibitors of CAIX have been developed [26,27], and cell-surface proteins such as CAIX are attractive targets because the delivery of CAIX inhibitors is not limited by penetration of adequate concentrations of inhibitor into the cell. However, it is worth noting that (as with any systemic therapeutic) diffusion and delivery of CAIX inhibitors to hypoxic cells in a poorly vascularized tumour microenvironment is an essential consideration. Nevertheless, the strong link between CAIX expression and tumour cell hypoxia provides support for therapeutically targeting CAIX.

LOX is a hypoxia-induced secreted enzyme that cross-links collagens and elastin in the extracellular matrix [28,29]. LOX increases breast tumour cell migration and invasion [18,30,31], and was recently shown to modulate the recruitment of bone marrow-derived cells to distant metastatic target organs [19]. The accumulation of bone marrow-derived cells in distant organs helps to create the so-called "pre-metastatic niche" that is thought to represent fertile regions of tissue that promote the growth of metastatic tumour foci [32,33]. The influence of hypoxia-induced LOX on two distinct steps in the metastatic process highlights LOX as an attractive therapeutic target for the reduction of metastatic disease. The inhibition of LOX using β-aminoproprionitrile (βAPN) or anti-LOX antibodies is effective in pre-clinical models [18,19], and the development of specific small molecule inhibitors of LOX is currently underway.

C-X-C chemokine receptor type-4 (CXCR4) is expressed on a range of normal cell types and is involved in several physiological processes, including hematopoiesis, angiogenesis, leukocyte trafficking, and leukocyte homing [34]. CXCR4 is hypoxia-inducible [35] and CXCR4 expression on metastatic tumour cells induces homing of disseminated tumour cells to specific tissues that express high levels of the CXCR4-specific ligand stromal cell-derived factor-1α (SDF-1α/CXCL12) [36]. The importance of CXCR4 in tissue-specific metastasis has resulted in the development of several inhibitors of the CXCR4/SDF-1α axis [37,38]. While the importance of CXCR4 in several normal tissue processes may complicate the therapeutic inhibition of this receptor, CXCR4 remains an extremely attractive therapeutic target to disrupt tumour metastasis.

The established role of several hypoxia-induced genes in promoting metastatic tumour cell dissemination and growth of metastatic tumour foci underscore the therapeutic potential of inhibiting the activity of hypoxia-induced proteins to reduce tumour metastases. Inhibition of HIF-1α, CAIX, LOX, or CXCR4 to reduce the development and growth of tumour metastases represent rational therapeutic strategies to disrupt the metastatic process. A complimentary strategy is to utilize compounds that have been designed to specifically kill cells at low oxygen tensions, and there are several hypoxia-activated cytotoxins with exciting potential to treat tumour metastases.

### Hypoxia-activated cytotoxins

Hypoxic tumour cells can be directly targeted using pro-drugs that are metabolically reduced to cytotoxic agents in cells at low oxygen tensions [39,40]. The use of hypoxia-activated cytotoxins to treat metastatic disease is supported by the detection of hypoxic cells in metastatic tumour foci in a number of pre-clinical tumour models. Some groups have shown that micrometastases smaller than approximately 1 mm^3 ^can be hypoxic [41-46], while other groups have found hypoxic tumour cells develop in metastases as they grow larger than 2 to 3 mm^2 ^in diameter [47]. Clinical data regarding the hypoxic fraction of metastatic tumours are lacking, due in large part to infrequent biopsying and subsequent immunohistochemical analysis of hypoxic cells in tumour metastases. It is worth noting, however, that relatively large clinical metastases can contain hypoxic tumour cells as evidenced by uptake of the radiolabeled hypoxia marker ^18^F-EF5 assessed by positron emission tomography (PET) [48]. Taken together, these pre-clinical and clinical observations indicate that microscopic and macroscopic metastatic tumour foci can contain hypoxic tumour cells that are, therefore, directly targetable using hypoxia-activated cytotoxins.

Tirapazamine (TPZ) [49] is a hypoxia-activated cytotoxin that has advanced the furthest in a clinical setting. In addition to numerous pre-clinical studies demonstrating efficacy of TPZ in combination with ionizing radiation and chemotherapy [39,40], TPZ has also been shown to reduce metastases when used as a neoadjuvant to radiation therapy in mice [50]. After several encouraging Phase I and II clinical trials, tirapazamine was moved into Phase III trials. However, several Phase III trials have reported unacceptable toxicity levels and conflicting results with respect to the benefit of incorporating TPZ into standard therapy regimens [51]. Moreover, a large Phase III clinical trial using TPZ in combination with cisplatin-based chemoradiotherapy did not report improved overall survival or relapse-free survival with TPZ [52], although several important issues have been raised with respect to this trial [53,54] that serve as important lessons for the future clinical testing of hypoxia-activated cytotoxins. A common issue with the Phase III TPZ trials is the failure to pre-select patients with significant numbers of hypoxic cells in their tumours for treatment with the hypoxia-activated drug [55]. There are several methods for detecting and quantifying hypoxic tumour cells that can be applied in the clinic, and restricting the use of hypoxia-activated cytotoxins to patients with hypoxic cells in their tumours is critical to properly evaluate the therapeutic potential of these agents.

Another hypoxia-activated cytotoxin with encouraging activity is the alkylaminoanthraquinone N-oxide AQ4N [56,57]. AQ4N has pre-clinical activity in the treatment of primary tumours, and has been tested in several Phase I/II trials [40,58-60]. There is also some pre-clinical evidence that AQ4N can reduce metastasis [61] although it is not clear if the drug reduces metastatic dissemination by affecting the primary tumour and/or directly targets hypoxic cells in the tumour metastases. The 2-nitroimidazole phosphoramidate mustard prodrug TH-302 is another hypoxia-activated cytotoxin with impressive pre-clinical activity [62] that has recently completed a Phase I trial [63]. Although the effect of TH-302 on tumour metastases has not yet been reported, TH-302 remains a promising therapeutic for targeting hypoxic tumour cells.

The 3,5-dinitrobenzamide-2-mustard PR-104 is an emerging hypoxia-activated pre-prodrug with intriguing therapeutic potential [64,65]. PR-104 recently completed a Phase I clinical trial [66] and is currently being tested in Phase II trials. PR-104 could target tumour metastases due to the presence of hypoxic cells in the metastatic tumour foci and/or the generation of cytotoxic PR-104 metabolites by the hypoxia-independent enzymatic activity of intracellular α-ketoreductase 1C3 [65] expressed by many tumour cell types. Regardless, PR-104 has potential as a hypoxia-activated cytotoxin that targets metastatic tumour cells in addition to cells in the primary tumour. Importantly, both TH-302 and PR-104 display pre-clinical activity when used as single agents, which sets these compounds apart from earlier generation hypoxia-activated cytotoxins (such as TPZ) which demonstrate anti-tumour activity only when used in combination with radiation or chemotherapy.

### Patient selection for therapy designed to target hypoxic tumour cells

There are several instances where using therapeutic strategies to target or inhibit the activity of hypoxic tumour cells to overcome metastasis would be clinically beneficial. Importantly, the detection and quantification of hypoxic cells in a primary (and metastatic) tumour must be used to identify patients most likely to benefit from therapies designed to target or inhibit hypoxic tumour cells to treat metastases. There are several methods used to detect and quantify hypoxia in solid tumours ranging from physical pO_2 _probes inserted into tumours, to evaluating the expression levels of hypoxia-induced proteins, to administering compounds that bind in hypoxic tumour cells (for example, pimonidazole [67] or EF5 [68]) that are quantifiable by immunohistochemistry or PET (for example, ^18^F-EF5 [48]). In addition, when considering inhibitors of CAIX, LOX, or CXCR4, selection of patients should be based on examination of expression levels of these target genes in tumor biopsies or by PET imaging based on tumour retention of radiolabeled antibodies (for example, against CAIX). Patients presenting with overt metastatic disease could be selected for hypoxia-based therapy based on the level of hypoxia in their primary tumours and/or tumour metastases for direct targeting of hypoxic tumour cells by hypoxia-activated cytotoxins and to disrupt the metastatic process by inhibition of hypoxia-induced proteins.

In patients without obvious (detectable) tumour metastases at the time of presentation, the presence of hypoxic cells in their primary tumour would suggest the patient is more likely to have undetected disseminated tumour cells in their system and/or may be at higher risk for developing metastatic disease after treatment of their primary tumour [2-4]. These patients would likely benefit from the incorporation of standard systemic chemotherapy into the treatment regimen prescribed for their primary tumour in order to target potential subclinical metastatic disease, particularly if hypoxia-activated cytotoxins are included to target hypoxic cells in the primary tumour (and micrometastases). Concurrent inhibition of hypoxia-induced proteins would help to prevent the further dissemination of metastatic tumour cells and to limit the development and growth of subclinical tumour metastases. Patients that experience loco-regional relapse of their primary tumour after radiation therapy commonly develop metastatic disease, and recurrent tumours tend to be hypoxic. The potential for long-term inhibition of hypoxia-induced proteins in patients after primary tumour treatment to prevent the subsequent development and growth of tumour metastases is unknown, although the importance of proteins, such as LOX and CXCR4, in a number of normal physiological processes may preclude the long-term use of LOX or CXCR4 inhibitors as prophylactics against metastatic disease.

## Conclusions

Rational design of therapeutic strategies to overcome metastatic disease based on targeting hypoxic tumour cells and/or inhibiting the hypoxia-induced proteins that influence tumour metastasis holds great promise for improving the treatment of metastatic cancer. Pre-clinical and clinical therapeutic strategies to overcome metastatic disease based on the presence of hypoxic tumour cells in primary and metastatic tumours should be pursued to address this important issue.

## Abbreviations

βAPN: β-aminoproprionitrile; CAIX: carbonic anhydrase-9; CXCR4: C-X-C chemokine receptor type-4; HIF: hypoxia-inducible factor; LOX: lysyl oxidase; PET: positron emission tomography; pO_2_: partial pressure of oxygen; SDF-1α: stromal cell-derived factor-1α; TPZ: tirapazamine.

## Competing interests

The authors declare that they have no competing interests.

## Authors' contributions

KLB and SD drafted and approved the final version of the manuscript.

## Pre-publication history

The pre-publication history for this paper can be accessed here:

http://www.biomedcentral.com/1471-2407/11/504/prepub
